# How Cells Handle DNA Breaks during Mitosis: Detection, Signaling, Repair, and Fate Choice

**DOI:** 10.3390/cells8091049

**Published:** 2019-09-07

**Authors:** Ruth Thompson, Rachel Gatenby, Samuel Sidi

**Affiliations:** 1 Department of Oncology and Metabolism, University of Sheffield Medical School, Beech Hill Road, Sheffield S10 2RX, UK; 2 Department of Medicine, Division of Hematology and Medical Oncology, The Tisch Cancer Institute, Icahn School of Medicine at Mount Sinai, New York, NY 10025, USA; 3 Department of Cell, Developmental and Regenerative Biology, The Graduate School of Biomedical Sciences, Icahn School of Medicine at Mount Sinai, New York, NY 10025, USA; 4 Department of Oncological Sciences, Icahn School of Medicine at Mount Sinai, New York, NY 10025, USA

**Keywords:** DNA damage, DNA repair, mitosis, cancer, cell fate

## Abstract

Mitosis is controlled by a complex series of signaling pathways but mitotic control following DNA damage remains poorly understood. Effective DNA damage sensing and repair is integral to survival but is largely thought to occur primarily in interphase and be repressed during mitosis due to the risk of telomere fusion. There is, however, increasing evidence to suggest tight control of mitotic progression in the incidence of DNA damage, whether induced in mitotic cells or having progressed from failed interphase checkpoints. Here we will discuss what is known to date about signaling pathways controlling mitotic progression and resulting cell fate in the incidence of mitotic DNA damage.

## 1. Introduction

The cell cycle is tightly regulated throughout and this is especially apparent in mitosis. It is essential that nothing should go wrong at this point as chromosome mis-segregation from abnormal mitosis can lead to cancer and other serious diseases. Yet core DNA damage sensing and repair machineries are essentially suppressed during mitosis, presumably as a means to prevent telomere fusion [1,2]. There is, however, increasing evidence to suggest tight control of mitotic progression in the incidence of DNA damage, whether induced in mitotic cells [3,4,5], having progressed from “unfinished business in S phase” [6], or from failed earlier checkpoints. This review will discuss what is known to date about control of mitosis in the event of DNA damage (largely focusing on DNA double-strand breaks) and crosstalk between the DNA damage response (DDR), the spindle assembly checkpoint (SAC), and resulting control of cell fate.

DNA double-strand breaks are one of the most deleterious types of DNA damage. The most likely repair mechanisms during mitosis are the error-prone nonhomologous end joining (NHEJ), alternative end joining (Alt-EJ), and the single-strand annealing (SSA) pathways. In addition to this, extension of mitosis could cause chromosomal translocations, telomere fusions, and aneuploidy [1,7]. For this reason, it has been proposed that cells with damaged DNA progress through mitosis with similar kinetics to unperturbed cells and then initiate the DDR and begin repair in G1 phase, rather than facilitate a response in mitosis [8]. However, there is increasing evidence for the existence of a mitotic DNA damage checkpoint. Experiments in yeast have shown the existence of a mid-anaphase DNA damage checkpoint [9] which is dependent on the yeast homologs of securin and separase (Pds1 and Esp1, respectively) [10]. A more recent paper demonstrated that along with the canonical SAC proteins Mad1, Mad2, Bub1, Bub3, and Cdc20, the DDR proteins ATM and ATR are required for mitotic arrest following DNA damage [3]. However, unlike the standard SAC which is activated by incorrect microtubule attachment, a functional kinetochore is not required for DNA-damage-induced mitotic arrest [3]. There are many evolutionary differences between yeast and human mitoses, such as yeast cells using the ancient “closed” mitosis where the nuclear envelope does not disassemble prior to mitosis and may play some role in chromosome segregation [11]. However, whilst there is no direct evidence that this DNA-damage-induced SAC is conserved in higher eukaryotes, there is data demonstrating the involvement of the DDR checkpoint effectors ATM and ATR in the SAC in Xenopus, chicken [12], and human cells [13]. 

Whilst the existence of a SAC-dependent DNA damage checkpoint has not been demonstrated in human cells, there is increasing evidence that DNA damage can result in delayed mitotic transit and even reversal of mitotic progression. Cells exposed to radiation in early to mid-prophase have been shown to be able to decondense their chromosomes and return to G2 [4]. Smits et al. demonstrated that mitotic progression in U2OS cells after release from nocodazole is slower when cells are exposed to DNA damaging agents than in untreated cells [14] and it has been shown that p53-deficient cancer cells which progress into mitosis with damaged DNA following treatment with aphidicolin arrest in metaphase for up to 10 hours before ultimately dying without exiting mitosis [15]. Mikhailov et al. subsequently published that in several human cell types, mitotic cells treated with high doses of radiation, Adriamycin, or a topoisomerase II inhibitor all demonstrate prolonged mitosis [5]. Contrary to the mitotic DNA damage checkpoint in yeast observed by Kim and Burke [3], this DNA-damage-induced mitotic delay is not dependent on ATM but is dependent on the spindle assembly checkpoint, which is consistent with unpublished data from our lab [5]. Mad2 was identified as being present on at least one kinetochore of each of five metaphase arrested cells analyzed postirradiation and the authors concluded that extensive damage to kinetochores was leading to activation of the spindle checkpoint. Giunta et al. claim that there was no difference in mitotic transit following DNA damage of mitotic cells but their data are not fully consistent with this and do seem to show a small population of cells which do not progress out of mitosis after irradiation (IR) [8]. Consistent with these findings, we observed delayed progression through mitosis following treatment with radiation and a Chk1 inhibitor [16], indicating that, as with damage induced directly in mitotic cells, damage induced in interphase which persists into mitosis due to dysfunctional interphase checkpoints also results in mitotic arrest [16]. We believe these data suggest substantial crosstalk between the DDR and the SAC in mitosis and hypothesize that the DDR senses mitotic DNA damage and activates the SAC. The question of whether or not this is dependent on specific damage to the kinetochores as suggested by Mikhailov et al. [5] requires further testing.

## 2. DNA Damage Signaling in Mitosis

In the last 10 years, it has emerged that, contrary to popular belief, there is at least a partial DNA damage response in mitotic cells [17,18]. When mitotic cells encounter double-strand breaks, it has been found that the MRN complex, ATM, DNA-PK, H2AX, and MDC1 are activated, as in the interphase checkpoints [7,8]. At this point, in the interphase checkpoints, MDC1 activates repair mechanisms by firstly recruiting the E3 ligase RING-finger protein 8 (RNF8), which enables the poly-ubiquitination of H2A and H2AX histones [8,19]. RNF168, another E3 ligase, is stimulated by the actions of RNF8 and further ubiquitinates these histones [7,20]. This ubiquitination leads to chromatin changes, recruiting 53BP1 and BRCA1 for NHEJ and homologous recombination, respectively [8,19]. However, these events are all repressed in mitosis [8].

The mitotic kinases, CDK1 and Plk1, negatively regulate the DDR in mitosis at various points [18]. CDK1 phosphorylates and inhibits RNF8 [1], therefore preventing downstream signaling, and further inhibits NHEJ by inactivating 53BP1 via phosphorylation at T1609 and S1678. Plk1 also phosphorylates 53BP1 at S1618 [1,7] and interacts with it, allowing Plk1 to inhibit the cell cycle kinase Chk2 via phosphorylation at multiple sites, preventing the DDR. The suppression of Chk2 is enhanced by the lessened ability of ATM to activate the kinase during mitosis [18]. Finally, Plk1 acts to prevent the ATR-Chk1 axis. ATR activation of Chk1 requires the adaptor protein claspin, which Plk1 targets for degradation by β-TrCP-SCF [21,22,23]. This results in Chk1 suppression, inhibiting the DDR. It is also important to note that many DNA damage checkpoint and repair proteins are subject to a vast array of mitotic-specific post-translational modifications.

Despite these various inhibitions, there is evidence for at least minimal DNA repair in mitosis. Terasawa et al. demonstrated that treatment of mitotic cells with the topoisomerase II inhibitor etoposide led to the accumulation of DNA double-strand breaks (DSBs) and dicentric chromosomes [20]. Whilst most cells with DSBs progressed to and were repaired in G1, a small proportion of DSBs were repaired in mitosis [20]. This is evidence against the supposition that there is no DNA repair in mitosis. The study went on to show that the NHEJ protein XRCC4 is phosphorylated specifically in mitosis and that CDK2 and Plk1 activity was required for this phosphorylation. 

Cells expressing an unphosphorylatable mutant form of XRCC4 were found to have elevated levels of NHEJ in mitotic cells, leading to increased numbers of chromatin bridges [20]. First described in 1941 [24], chromatin bridges (also known as anaphase bridges) occur when DNA from two chromosomes or chromatids is fused together either through incorrect DNA repair or telomere fusion [25]. As previously mentioned, it has been proposed that this is the reason for restriction of repair in mitosis and there is evidence that inaccurate repair in mitosis can lead to DNA bridges [1,20,26]. In addition to DNA breaks, these chromatin bridges can be formed by consistent chromatid cohesion [27] and by telomere shortening [28]. 

A very recent paper has demonstrated that MDC1 foci, which form at DNA breaks in mitosis, recruit TopBP1, which then forms filamentous structures [29]. The authors propose that these filamentous structures tether DSBs and allow proper segregation of broken chromosomes until repair can be carried out in G1 [29]. Interestingly, a similar observation was made in *Drosophila* cells whereby acentric chromosomes arising from DNA breaks are tethered to the rest of the chromosome by DNA decorated with various mitotic proteins including Plk1, Aurora B, and the spindle assembly checkpoint protein BubR1 [30]. 

It has been shown that DNA lesions induced by replication stress can be carried over from mitosis to G1 when compartmentalized and protected by “nuclear bodies” containing certain genome maintenance factors including 53BP1, MDC1, pATM, BRCA1, NBS1, and TopBP1 [31]. 53BP1 nuclear bodies seem to form particularly at “chromosomal fragile sites” (CFSs), which are areas prone to lesions in mitosis following replication stress in S phase [32]. These fragile sites have been shown to lead to “ultrafine DNA bridges”, which are the most common form of unresolved mitotic structure and maintain physical links between sister chromatids in anaphase and are characterized by PICH and BLM staining [33,34]. It has been shown that replication stress activates DNA repair synthesis in mitosis [35]. This mitotic DNA repair synthesis (termed MiDAS) requires the recruitment of Mus81 to CFSs and leads to POLD3-dependent synthesis [35]. TopBP1 foci form in mitosis at sites of unscheduled DNA synthesis and persistent TopBP1 foci transition into 53BP1 nuclear bodies [36]. Below we expand upon the model proposed by Leimbacher et al., incorporating both members of the spindle assembly checkpoint and proteins shown to localize to ultrafine DNA bridges.

**Figure 1:**
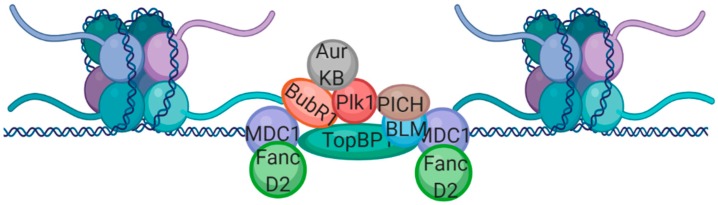
Acentric chromosomes resulting from DNA breaks or replication stress could lead to chromosomal instability if incorrectly segregated. We propose that multiprotein complexes made of DDR and SAC proteins form to tether broken chromosome fragments and facilitate accurate chromosome segregation. Image created using BioRender.

Leimbacher et al. demonstrate that MDC1 recruits TopBP1 to DNA double-strand breaks specifically in mitosis and TopBP1 then forms filamentous structures which bridge MDC1 foci [29]. BubR1, along with Plk1, has also been shown to bridge gaps and allow for proper chromosome segregation in Drosophila [30]. As BubR1 and MDC1 have been shown to interact in human cells in mitosis [13], we propose that these DNA tethers are one and the same, suggesting further crosstalk between the spindle checkpoint and DNA repair pathways. BubR1 and Plk1 have been found to interact in human cells [37] and systematic studies into the human interactome also indicate that TopBP1 interacts with Plk1 [38]. BLM, which has been demonstrated to localize to DNA bridges (both DAPI stained and ultrafine) [34], also interacts with TopBP1 [39] and the Plk1 Interacting Checkpoint Helicase (PICH) [33] is another protein often associated with ultrafine DNA bridges [34]. Another important protein which is involved both in DSB repair in interphase and mitotic UFB resolution is Rif1. Rif1 acts downstream of ATM/53BP1 to inhibit resection of broken DNA ends, thus inhibiting HR and promoting NHEJ [40] However, it also localizes to UFBs in mitosis in a PICH-dependent manner and is required for timely UFB resolution [41]. Finally, FancD2, which flanks ultrafine bridges (much like MDC1 flanks TopBP1 filamentous structures), interacts with MDC1 [42]. From these collective observations, we propose that the broken DNA “tethers” described by Leimbacher and Royou [29,30] and the ultrafine bridges resulting from replication stress [34] may in fact all be working together in a similar pathway and that multiprotein structures form at the sites of DNA breaks in mitosis, to allow for faithful segregation of chromosome fragments prior to repair in G1 (Figure 1). 

## 3. Crosstalk between the SAC and DDR

The spindle assembly checkpoint (SAC) is the main checkpoint in mitosis and acts to ensure faithful segregation of chromosomes to avoid aneuploidy. The SAC becomes active as a cell enters mitosis and remains so until each chromosome is properly attached to the spindle apparatus [43]. The main component of the SAC is the mitotic checkpoint complex (MCC), which consists of the proteins BubR1, Bub3, and Mad2 [43,44]. The MCC, whilst active, binds the cell division protein Cdc20. Cdc20 is required for the activation of the Anaphase-Promoting Complex (APC), which promotes anaphase through the degradation of securin, which holds sister chromatids together [45]. Once the SAC is satisfied, the MCC releases Cdc20, which can then bind and activate the APC and initiate anaphase [43]. The SAC is not currently accepted to be a DNA damage checkpoint but there is evidence emerging to indicate extensive crosstalk between the known DDR and the SAC.

**Figure 2:**
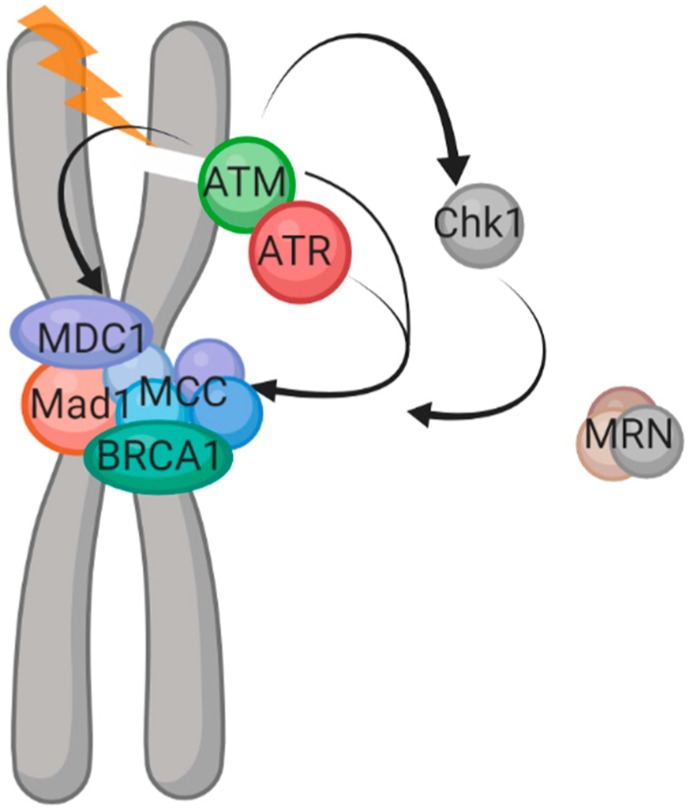
Summary of documented interplay between the DDR and SAC. DDR effectors ATM/ATR and downstream Chk1 have all been demonstrated as playing a role in SAC control, both in the unperturbed cell cycle and cells exposed to DNA damage.

There is mounting evidence for a role for the DNA damage sensors ATM and ATR in the control of the SAC. In 2008, Kim and Burke demonstrated that in yeast cells, DNA damage which progresses through from interphase activates the SAC and this is dependent on the ATM and ATR homologs, Mec1 and Tel [3]. A similar observation was made in *Xenopus* mitotic extracts and somatic cells whereby DNA restriction enzymes were added to induce DNA breaks in mitosis and this led to a prolonged mitosis dependent on ATM and ATR [46]. Whilst the work by Mikhailov et al. demonstrates that loss of ATM activity via caffeine does not abrogate DNA-damage-induced mitotic arrest in human cells [5], more recently, ATM has been shown to regulate the spindle assembly checkpoint in unperturbed human cells through the dimerization of Mad1 (Figure 2) [47] and also via the recruitment of the DNA damage repair protein MDC1 to kinetochores [13]. It was found that ATM, ɣH2AX, and MDC1 are required for kinetochore localization of the MCC proteins Mad2 and Cdc20 and that the absence of these proteins leads to an ineffective SAC and more rapid mitotic progression [13] (Figure 2). A role for the fission yeast homolog of MDC1 (Mdb1) has been proposed in mitotic spindle function as it was found to localize to spindles and loss of the protein sensitized cells to spindle poisons [48], however, the exact role of MDC1 in mitosis is under dispute as another group has purported that MDC1 loss leads to a prolonged mitosis [49]. They hypothesize that MDC1 promotes anaphase via an interaction with the anaphase-promoting complex previously identified by the Goldberg group [50]. Later work by the Goldberg group, however, as discussed above, revealed the opposite effect whereby cells with reduced MDC1 transitioned through mitosis more rapidly [13]. 

Chk1, a downstream kinase of ATM and ATR in the canonical DNA damage response, has been suggested to play a role in the spindle checkpoint in human cells [51] supposedly though Aurora B and BubR1 (Figure 2). This is consistent with findings in *Drosophila*, where DNA damage led to delayed metaphase/anaphase transition which was dependent on the Chk1 homolog [52], but contrary to both our work in human cells and Kim and Burke’s work in yeast cells whereby the DNA-damage-induced SAC was observed in cells where Chk1 had been either deleted or inhibited in order to push cells through the interphase checkpoints with DNA damage [3,16]. There is also mounting evidence for other DDR proteins playing a role in centromere stability and the SAC. Immunofluorescence and chromatin IP studies have shown BRCA1 at centromeres [53] and a recent study has demonstrated an important role for the MRN complex (essential for the initial processing of double-strand breaks prior to repair) in mitosis [54] (Figure 2). In mitotic cells, MRN was found to interact with MMAP and colocalize in mitotic spindles. Cells deficient for MRN or MMAP were found to have abnor mal spindle dynamics and chromosome segregation [54]. 

## 4. Crosstalk between the SAC and Cell Death

Mitotic catastrophe, as defined in 2012 by the International Nomenclature Committee on Cell Death, is an “intrinsic oncosuppressive mechanism that senses mitotic failure and responds by driving a cell to an irreversible antiproliferative fate of death or senescence” [55]. From this definition, cell death resulting from improper mitosis can occur either within mitosis or following G1. The signaling pathways which result in mitotic catastrophe are largely unknown, however, we and others have demonstrated crosstalk between the SAC and the apoptotic machinery which appears to be upregulated in response to excessive DNA damage. 

It has been demonstrated that SAC function is required for mitotic catastrophe when cells treated with DNA damaging agents enter mitosis with unrepaired DNA lesions [15]. As previously mentioned, these cells were shown to exhibit prolonged mitosis before subsequently dying in metaphase [15]. Cells retained BubR1 at the centromeres and did not activate the APC. This mitotic arrest and ultimate catastrophe were shown to be dependent on SAC proteins BubR1 and Mad2 as cells with reduced levels of these proteins were found to escape mitotic death and divide abnormally [15].

The PIDDosome (PIDD-RAIDD-caspase-2) is an “orphan” caspase activation platform which results in apoptosis independent of the canonical apoptosome (caspase-9-activating) and DISC (caspase-8-activating) platforms in the intrinsic and extrinsic apoptosis pathways, respectively [56,57,58]. We have demonstrated that caspase-2 activation via the PIDDosome is required for apoptosis following irradiation in the absence of Chk1 [59,60,61]. Interestingly, we discovered that the SAC protein BubR1 directly binds PIDD and inhibits PIDDosome formation in mitosis [16]. This interaction appears to be upregulated following treatment with irradiation, indicating that the SAC and the apoptotic machinery communicate in mitosis in response to DNA damage. The inhibition of PIDDosome formation in mitosis is short-lived and once cells exit mitosis, the PIDDosome assembles and apoptosis is initiated. We propose that BubR1 and PIDD communicate in mitosis to (1) restrict apoptosis in mitosis and (2) determine cell fate for the following G1 if damage is too severe [16,62]. 

## 5. The SAC and Senescence

**Figure 3:**
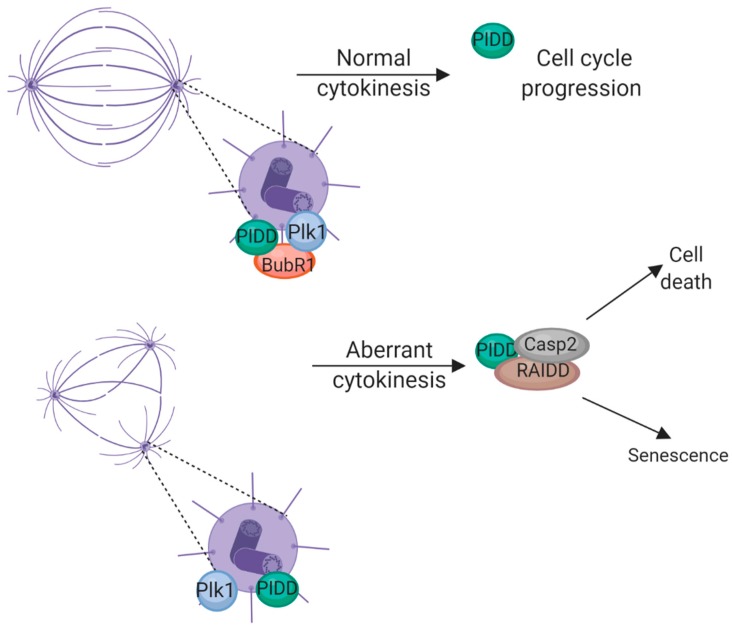
Interaction of BubR1 and PIDD at centrosomes. In the incidence of supernumerary centrosomes, PIDD interacts with RAIDD and assembles to PIDDosome resulting in either cell death or senescence depending on p53 status. Image created using BioRender.

There is a p53 checkpoint in G1 which detects incorrect centrosome numbers resulting from aberrant cytokinesis [63,64]. More recent evidence suggests this is via the PIDDosome [65]. Fava et al. demonstrate that the PIDDosome is activated in G1 in response to supernumerary centrosomes and this leads to p21-dependent cell cycle arrest via caspase-2-dependent MDM2 cleavage and subsequent p53 stabilization [65]. BubR1 has been demonstrated to localize to centrosomes in interphase in a Plk1-dependent manner [37]. BubR1 then prevents centrosomal amplification via inhibition of Plk1 activity [37]. Fava et al. identified PIDD1 foci at centrosomes and we have also observed phosphorylated T788 PIDD foci at the centrosomes of anaphase cells via immunofluorescence [Thompson and Sidi, unpublished observations]. These observations, together with our published data that BubR1 interacts with PIDD in mitosis to inhibit RAIDD binding and thus PIDDosome formation [16], led us to hypothesize that BubR1 and PIDD communicate through mitosis and into the subsequent G1 to detect aberrant centrosomes (Figure 3). 

Plk1 and BubR1 localize to centrosomes primarily in interphase and this localization was not observed in mitotic cells [37]. From these observations, we hypothesize that Plk1 recruits BubR1 to centrosomes in interphase and BubR1 in turn recruits PIDD in mitosis. As cells enter anaphase and BubR1 is lost from the centrosomes, PIDD is free to interact with RAIDD and activate either senescence or death pathways (Figure 3). How PIDD specifically detects aberrant numbers of centrosomes is unclear as we and Fava et al. detected PIDD foci at centrosomes of anaphase cells with normal centrosomes. It is possible that supernumerary centrosomes lead to BubR1 being titred out so there is insufficient BubR1 molecules to outcompete RAIDD binding to PIDD. Our data that p53 null zebrafish and p53 mutant cell lines undergo PIDDosome-mediated cell death following SAC failure due to loss of BubR1 [16] and Fava et al.’s observations that there is a p53-dependent, PIDDosome-mediated cell senescence pathway activated by cytokinesis failure have led us to the hypothesis that p53 is acting as a cell-fate “switch” with p53-positive cells undergoing senescence following cytokinesis failure and p53 null cells undergoing cell death (Figure 3).

### DNA Damage, the SAC, and Inflammation

It is no new discovery that ionizing radiation induces an inflammatory response in human cells [66], however, very recently, the mechanism for this has begun to emerge. It was discovered that the delayed immune response resulting in shrinkage of abscopal tumors (those not being directly targeted) was dependent on DNA DSB and coincided with the appearance of aberrantly shaped nuclei and micronuclei [67,68]. CDK1 and Plk1 inhibitors which blocked mitotic entry blocked the appearance of micronuclei and subsequent inflammatory response and the major cytosolic DNA sensor cyclic GMP-AMP synthase (cGAS) was found to localize to micronuclei postmitosis [67]. Chromosome instability induced by overexpression of the kinesin-13 protein family member Kif2b (microtubule-dependent motor required for spindle assembly) and MCAK (required for tubulin depolymerisation) which leads to higher levels of micronuclei due to chromosome mis-segregation was found to lead to rapid metastasis and poor prognosis in mice [69]. Interestingly, overexpression of Mad2 was found to partially rescue this phenotype [69]. The cGAS-STING axis has been demonstrated as being essential for both cell senescence [70,71,72] and cell death via apoptosis [73,74] and necrosis [75], providing another route by which DNA damage can lead to aberrant mitosis and altered cell fate.

## 6. Conclusions

Here we have reviewed the literature around DNA damage recognition and repair in mitosis. While popular belief is that DNA repair is largely suppressed in mitosis [1,7,8], we and others have demonstrated that cells respond to mitotic DNA damage by transitioning through mitosis more slowly [5,14,15,16]. Furthermore, despite DNA repair pathways being inhibited at various points in mitosis via mitotic kinases Plk1 and CDK2 [18,20,21], the repair proteins are involved in elaborate complexes which tether broken chromosomes [29,30,34]. Furthermore, certain cell fate decisions appear to be instigated in mitosis, again even though not physically carried out until G1 [16,31,36,65]. These observations make a compelling case for the existence of a DNA damage checkpoint in mitosis, even if most DNA repair does not physically happen and cell fate is not fully determined until the next G1. As our data also indicates that cell death resulting from excessive DNA damage and loss of interphase checkpoints is initiated in G1, it suggests that cell fate, whilst possibly decided in mitosis, is not carried out until G1. The evidence provided by Kim and Burke that, in contrast to the standard SAC, a functional kinetochore is not required for the mitotic DNA damage checkpoint indicates that at least in yeast, these are two separate checkpoints (although admittedly they have considerable crosstalk). 

This review has identified several gaps in the field and identified where future work needs to be concentrated. A major question is how DNA damage is sensed in mitosis and how this in turn leads to a prolonged SAC. While there is ample evidence of crosstalk between the DDR and the SAC [3,13,46,51,54], it is unclear exactly how the DDR communicates with the SAC to instigate cell cycle arrest. We also provide evidence here for the SAC protein BubR1 playing a vital role in restriction of genomic instability resulting from broken chromosomes. Future work needs to determine whether the BubR1-coated filamentous structures identified by Royou et al. in *Drosophila* cells [30] are one and the same as the MDC1 and TopBP1 structures recently identified by Leimbacher et al. [29]. Furthermore, it remains to be seen whether these structures, like ultrafine bridges resulting from chromosome fragile sites, are also stained with PICH and BLM and whether these bridges are all processed in the same way once they progress into G1. Finally, more work needs to be carried out to uncover the precise mechanism by which supernumerary centrosomes activate the PIDDosome, how Plk1 and BubR1 are involved in this, and whether p53 is the only decider of cell fate in this event. All of these questions are vital in the understanding of how cells process DNA damage and thus for the development of DNA-damage-inducing anticancer drugs.
